# The clinical impact of galectin-8 in drug resistant breast cancer

**DOI:** 10.7150/jca.104000

**Published:** 2025-01-13

**Authors:** Yi-Chung Chien, Jia-Yan Wu, Chi-Chun Pang, Ruey-Hwang Chou, Yung-Luen Yu

**Affiliations:** 1 Institute of Translational Medicine and New Drug Development, China Medical University, Taichung 406040, Taiwan.; 2 Graduate Institute of Biomedical Sciences, China Medical University, Taichung 406040, Taiwan.; 3 Center for Molecular Medicine, China Medical University Hospital, Taichung 404327, Taiwan.; 4 The Ph. D. Program of Biotechnology and Biomedical Industry, China Medical University, Taichung 406040, Taiwan.; 5 Department of Medical Laboratory Science and Biotechnology, Asia University, Taichung 413305, Taiwan.

**Keywords:** galectin-8, breast cancer, drug resistant, prognosis

## Abstract

Breast cancer remains the leading cause of cancer-related mortality among women globally. A significant challenge in lowering breast cancer death rates is multidrug resistance. This resistance arises through various mechanisms, such as heightened drug efflux, improved DNA repair, escape from senescence, epigenetic modifications, tumor heterogeneity, alterations in the tumor microenvironment (TME), and the epithelial-to-mesenchymal transition (EMT). These factors collectively make overcoming drug resistance particularly difficult. Therefore, in this study, we analyzed data from The Cancer Genome Atlas (TCGA) and identified a novel gene, galectin-8, which plays a critical regulatory role in breast cancer progression. Gene Set Enrichment Analysis (GSEA) further revealed that galectin-8 is involved in modulating drug resistance in breast cancer. To validate this finding, we conducted a mass assay comparing drug-resistant triple-negative breast cancer (TNBC) cell lines with control groups. Our results demonstrated a significant increase in galectin-8 expression in the drug-resistant cells, with statistically significant differences observed. In addition, we found that reducing galectin-8 expression in drug-resistant cell lines not only reinstated the effectiveness of anticancer drugs but also suppressed tumor cell proliferation and migration. Therefore, our findings highlight the significant prognostic and therapeutic potential of galectin-8, emphasizing the importance of future research to explore targeted therapeutic strategies in breast cancer.

## Introduction

Breast cancer is the most frequently diagnosed malignancy in women worldwide, and it remains the leading cause of cancer-related deaths. Globally, over 2 million women are diagnosed with breast cancer each year, with mortality primarily driven by the emergence of resistance to conventional treatments 1. Despite advancements in therapeutic strategies such as surgery, radiation therapy, chemotherapy, and the development of targeted therapies, the success of these treatments is often limited by the development of multidrug resistance (MDR) 2, 3. MDR is a complex phenomenon that involves multiple mechanisms, including increased drug efflux through ATP-binding cassette (ABC) transporters, enhanced DNA repair capacity, epigenetic alterations, and changes in the tumor microenvironment (TME), all of which contribute to the survival of cancer cells even in the presence of cytotoxic agents 4. These resistance mechanisms are particularly problematic in aggressive subtypes of breast cancer, such as triple-negative breast cancer (TNBC), which lacks expression of hormone receptors and HER2, making it more difficult to treat with conventional therapies 5. As a result, overcoming MDR in breast cancer remains a critical challenge in improving patient outcomes.

One of the emerging factors implicated in tumor progression and resistance is galectin-8, a member of the galectin family of β-galactoside-binding proteins. Galectins are known to regulate a variety of biological processes, including cell adhesion, migration, immune response modulation, and apoptosis 6, 7. Galectin-8, in particular, has been shown to be overexpressed in several types of cancer, including breast cancer, and its presence is associated with poor clinical outcomes 8. Studies have revealed that galectin-8 plays a crucial role in promoting tumor growth and metastasis by modulating the tumor microenvironment. Galectin-8 can facilitate immune evasion by recruiting immunosuppressive cells, such as myeloid-derived suppressor cells (MDSCs) and regulatory T cells (Tregs), while simultaneously reducing the infiltration and activity of cytotoxic CD8^+^ T cells 9. This immune-suppressive environment allows cancer cells to evade immune surveillance and contributes to tumor progression. Additionally, galectin-8 is involved in the epithelial-to-mesenchymal transition (EMT), a key process that enables cancer cells to acquire invasive and metastatic properties, as well as resistance to chemotherapy 10, 11. Given these roles, galectin-8 is thought to be a significant player in the development of drug resistance in breast cancer.

Given its multifaceted roles in promoting tumor progression and immune evasion, galectin-8 has emerged as a potential therapeutic target in breast cancer. However, the specific role of galectin-8 in the development of drug resistance remains poorly understood. In this study, we aim to explore the expression levels of galectin-8 in drug-resistant breast cancer cell lines and investigate its potential role in promoting the MDR phenotype. By elucidating the molecular mechanisms by which galectin-8 contributes to drug resistance, we hope to identify new therapeutic strategies that target galectin-8 to overcome resistance in breast cancer. This could ultimately lead to improved treatment outcomes for patients with breast cancer, particularly those with drug-resistant tumors 12, 13. Furthermore, the study will evaluate the potential of galectin-8 as a prognostic marker, offering insight into its utility in predicting treatment responses and guiding therapy decisions in breast cancer management.

## Materials and Methods

### Cell lines, cell culture conditions, and drugs

All the cell lines utilized in this study were sourced from the American Type Culture Collection. Human TNBC cell lines MDA-MB-231 and MDA-MB-468 were cultured in DMEM/F12 (1:1) medium supplemented with 10% (v/v) fetal bovine serum (GIBCO). The BT-549 human TNBC cell line was grown in RPMI-1640, enriched with 10% (v/v) fetal bovine serum and 1% (w/v) penicillin/streptomycin. The non-tumorigenic mammary epithelial cell line MCF-10A was maintained in DMEM/F12 containing 1.05 mM CaCl2, 100 ng/mL cholera toxin, 5% (v/v) horse serum (Gibco), 10 μg/mL insulin, 100 U/mL penicillin, 100 μg/mL streptomycin, 20 ng/mL EGF (Sigma), and 500 ng/mL hydrocortisone. All cultures were maintained in a humidified incubator at 37°C with 5% CO_2_. To generate Tarceva-resistant MDA-MB-231 and BT-549 cells, we exposed them to increasing concentrations of Tarceva (Roche) over 6 months, starting at 5 μM and gradually increasing to 100 μM. After 8 months, resistant clones were established and maintained in 2 μM Tarceva.

### Comprehensive analyses of *LGALS8* (*galectin-8*) from The Cancer Genome Atlas (TCGA)

UALCAN stands as an intuitive and interactive web platform designed for the examination of cancer omics data, accessible at http://ualcan.path.uab.edu/index.html. It leverages TCGA level 3 RNA-seq alongside clinical data across 31 cancer types 14. In our research, the UALCAN tool was utilized to conduct analyses concerning the differential expression between tumor and normal tissues, as well as to assess the impact of *LGALS8* expression on the overall survival of HCC patients.

### To identify gene set enrichments of galectin-8 in TCGA database

The Cancer Genome Atlas (TCGA) and analogous projects have yielded invaluable tumor-associated genomic data. Despite several web-based platforms designed to enhance accessibility, certain analyses require prior bioinformatic expertise. Therefore, to identify gene set enrichments of galectin-8 in the TCGA database, we employed the Gene ENrichment Identifier (GENI, https://www.shaullab.com/geni). This tool is specifically designed to rapidly calculate correlations between the gene of interest and the entire transcriptome, then rank them against well-established biological gene sets 15.

### Mass Spectrometry (MS)-based proteomic identification

Proteins were digested into peptides using trypsin, followed by purification and loading onto a nanoLC C18 analytical column for separation. Peptides were ionized via electrospray ionization (ESI) and analyzed using tandem mass spectrometry (LC-MS/MS). The resulting spectra were processed using search algorithms like Sequest or Mascot to match observed mass-to-charge ratios with theoretical peptides. Post-processing identified peptides were further examined for biological function and modifications 16.

### MTT assay

We evaluated the ability of galectin-8 to promote the proliferation of breast cancer cells using the MTT assay. Cells were seeded into 96-well culture plates at a density of 3 × 10^3^/well. The cells were cultivated for an additional 24 h at 37ºC prior to the addition of 10% (w/v, final concentration) MTT in each well (Sigma; stock solution, 250 mg/mL in PBS). Following incubation for 2-4 h, the formazan crystals produced in each well were dissolved in DMSO (50μl/well), and the absorbance was measured at 570 nm in the Gen5 microplate reader (BioTeck). To quantify the relative viability of each cells, its averaged absorbance was normalized to that of the corresponding control cells.

### Gene knockdown with shRNAs

Gene knockdown was performed using specific shRNAs delivered via a lentiviral system, sourced from the National RNAi Core Facility (Academia Sinica, Taipei, Taiwan), following the provided protocol. The shRNA constructs targeting galectin-8 were TRCN0000057354 (#1) and TRCN0000419140 (#2), while the shRNA targeting luciferase (VOID), clone TRCN0000072244, was used as a negative control. The efficiency and specificity of the knockdown were validated according to previously published methods 17.

### Quantitative PCR (qPCR)

Total RNA was isolated using TRIzol reagent (Invitrogen) following the manufacturer's protocol. Primers were synthesized by Invitrogen, with the following sequences used for gene amplification: galectin-8 forward (5'-GCATGTTCCTAGTGACGCAG-3') and reverse (5'-GATCTCTTCCCGTCCCCATT-3'), ZEB1 forward (5'-ACTGCTGGGAGGATGACAGA-3') and reverse (5'-ATCCTGCTTCATCTGCCTGA-3'), ZEB2 forward (5'-AAAACCATGGCGTGGGTA-3') and reverse (5'-CAATAGCCGAGGCATCAAC-3') and GAPDH, as the internal control, forward (5'-ACCACAGTCCATGCCATCAC-3') and reverse (5'-TCCACCACCCTGTTGCTGTA-3'). Quantitative PCR (qPCR) was conducted as outlined in previous studies 17, 18, detection was performed using the LightCycler 480 system (Roche).

### Western blot analysis

The following antibodies and reagents were used in the experiments: anti-Galectin 8 (Abcam, Burlingame, 1:1000), anti-E-cadherin (Cell Signaling, 1:1000), anti-N-cadherin (GeneTex, 1:1000), anti-vimentin (Abcam, 1:1000), and anti-alpha-tubulin (GeneTex, 1:5000). Chemiluminescence detection was performed using a reagent from GE (Piscataway, NJ). The experimental protocol was conducted in accordance with previously published methods. 17.

### Transwell migration assays

Cell migration was assessed using Boyden chambers (Millipore, Billerica, MA). In summary, 5 × 10^4^ cells suspended in medium containing 1% FBS were plated onto membrane inserts with 8 μm pores. Following a 24-hour incubation, non-migrated cells on the upper side of the membrane were removed with a cotton swab. The cells that had migrated to the lower side were stained with Giemsa and counted under a light microscope at 100x magnification 19, 20.

### IncuCyte assays

Cells (1 × 10^5^ per well) were seeded into a 96-well plate and incubated at 37 °C for 24 hours. Wounds were created using the 96-well WoundMaker (Essen BioScience), followed by two washes with phosphate-buffered saline (PBS) to clear detached cells. Images of the wounds were automatically taken inside the CO_2_ incubator using IncuCyte software (Essen BioScience), with photos captured every 12 hours over a 24-hour period. Wound closure was evaluated by measuring wound confluence, and data were processed and analyzed with the IncuCyte software (Essen BioScience).

### Statistical analyses

Data analysis was performed using Prism 8 (GraphPad) and Microsoft Excel. Statistical comparisons were made using either a two-tailed Student's t-test or Fisher's exact test. A P-value of less than 0.05 was regarded as indicative of statistical significance.

## Results

### Expression levels of *LGALS8* (*galectin-8*) in clinical specimens and its impact on survival rates

**Figure 1:**
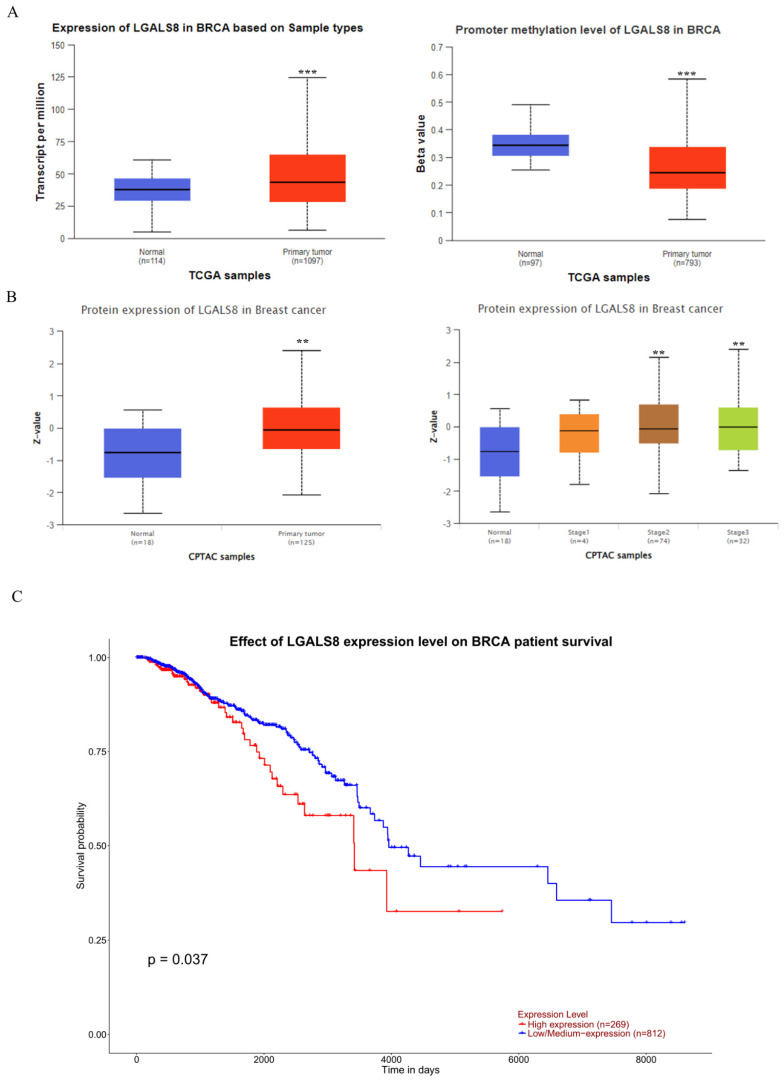
Expression levels of LGALS8 (galectin-8) in breast cancer and impact on survival. (A) Comparison of LGALS8 (galectin-8) RNA expression between normal and breast cancer tissues, along with galectin-8 promoter methylation levels. (B) Galectin-8 protein expression levels in breast cancer, showing higher expression in stage 2-3 breast cancer. (C) Kaplan-Meier survival analysis indicating that patients with higher galectin-8 expression have significantly shorter overall survival (OS). ** p < 0.01, *** p < 0.001.

To ascertain the role and significance of galectin-8 in breast cancer, we utilized UALCAN for analyzing breast cancer patient data from The Cancer Genome Atlas (TCGA) database. The study aimed to compare LGALS8 expression between normal and cancerous tissues, assess promoter methylation levels, and evaluate its impact on patient survival. First, we analyzed LGALS8 RNA expression levels across normal and breast cancer tissues. The results indicated significantly higher galectin-8 expression in breast cancer tissues compared to normal tissues (Fig. 1A). Additionally, we assessed galectin-8 promoter methylation levels, where beta values ranging from 0 (unmethylated) to 1 (fully methylated) were used. Hyper-methylation was defined with beta values between 0.7 and 0.5, and hypo-methylation with values between 0.3 and 0.25 (PMID: 29027401, 23291739). This differential methylation may influence galectin-8 expression in breast cancer. At the protein level, galectin-8 expression was quantified using Z-values, representing standard deviations from the median across breast cancer samples. The log2 spectral count ratio values from the CPTAC were normalized within each sample profile and across samples. Analysis revealed that galectin-8 protein levels were notably elevated in breast cancer tissues, particularly in stage 2 and stage 3 (mid-to-late stage) breast cancer (Fig. 1B). This confirmed that galectin-8 protein levels were elevated in breast cancer tissues, further supporting its role in cancer progression. Kaplan-Meier survival analysis demonstrated a significant correlation between galectin-8 expression and overall survival (OS). Patients with higher galectin-8 expression showed notably shorter OS compared to those with lower expression levels (Fig. 1C). This suggests that elevated galectin-8 expression is associated with poorer prognosis in breast cancer patients. Therefore, our findings underscore the potential significance of galectin-8 as a biomarker for breast cancer progression and prognosis. Its increased expression, coupled with promoter methylation changes, highlights its importance in aggressive tumor behavior and survival outcomes, making it a potential therapeutic target in breast cancer.

### Gene set enrichment analysis for comparison of high and low expressed galectin-8 from TCGA breast cancer

**Figure 2:**
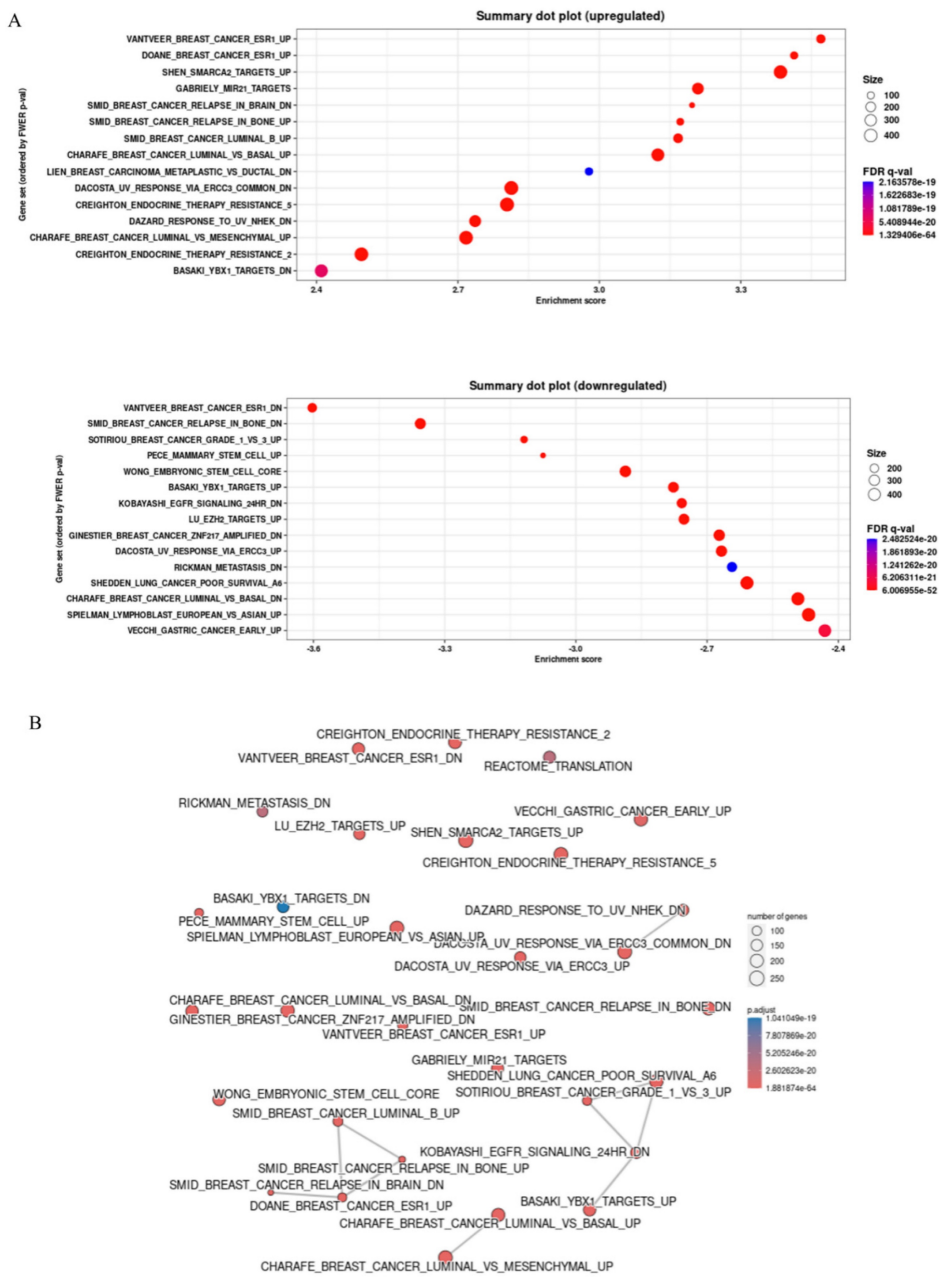

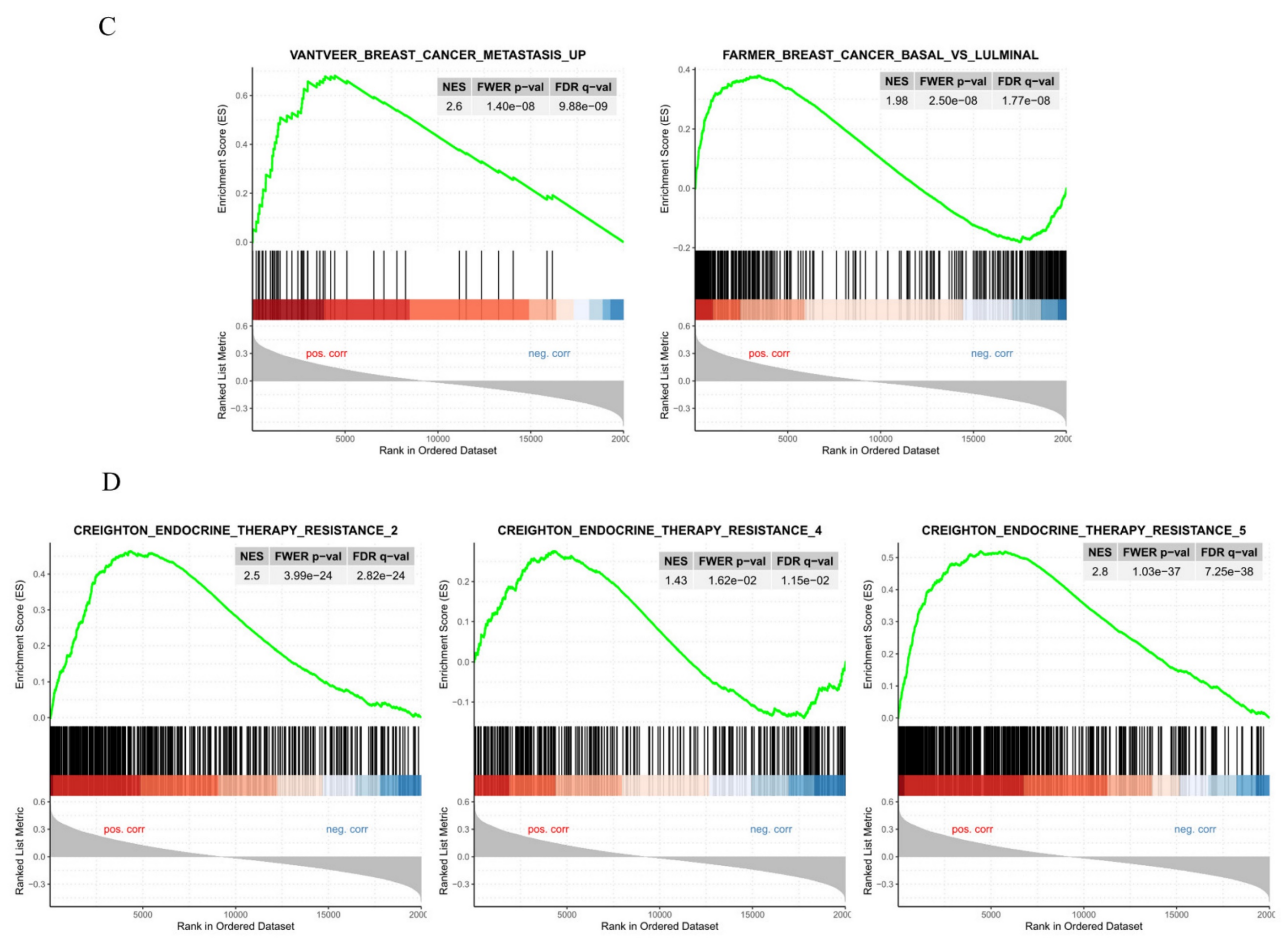
Gene set enrichment analysis for comparison of high and low expressed galectin-8 in TCGA breast cancer. (A) Dot plot showing the gene sets significantly upregulated and downregulated in the high galectin-8 expression group. The Y-axis lists the gene sets, with upregulated sets related to tumor progression and downregulated sets associated with normal cellular processes. (B) Correlation network illustrating the relationships between gene sets enriched in the high galectin-8 expression group. Strong associations are observed between pathways involved in EMT, immune evasion, and therapy resistance. (C) GSEA enrichment plot showing the positive association between high galectin-8 expression and EMT-related gene sets, indicating an increased metastatic potential in breast cancer cells. (D) GSEA enrichment plot showing the negative correlation between high galectin-8 expression and apoptosis-related pathways, suggesting that galectin-8 contributes to the inhibition of programmed cell death and therapy resistance.

The Gene ENrichment Identifier (GENI) platform was employed to compare gene expression profiles of high and low galectin-8 (LGALS8) expression groups, ranking them against known biological gene sets. This analysis was conducted using data from The Cancer Genome Atlas (TCGA) to identify pathways influenced by galectin-8 expression and its potential role in breast cancer progression and therapy resistance. The dot plot analysis revealed several gene sets that were significantly upregulated and downregulated in the high galectin-8 expression group (Fig. 2A). On the Y-axis, the upregulated gene sets were primarily associated with oncogenic processes such as tumor invasion, metastasis, and immune suppression, while the downregulated gene sets were involved in normal cellular functions. This result suggests that elevated galectin-8 expression enhances pathways that promote breast cancer aggressiveness and downregulates mechanisms that maintain normal cellular activity. A correlation network was constructed to depict the relationships between these gene sets (Fig. 2B). The network showed strong connections among gene sets related to EMT, immune evasion, and therapy resistance. These interconnections indicate that galectin-8 plays a central role in linking processes that drive tumor invasiveness, suppress the immune response, and enable cancer cells to resist treatments. Interestingly, the basal-like subtype of breast cancer, which is known for its poor prognosis and lack of hormone receptors, was highly enriched in the high galectin-8 expression group (Fig. 2C). This is a critical finding as the basal subtype is typically associated with higher rates of drug resistance and a more aggressive clinical course. The enrichment of basal subtype characteristics in the high galectin-8 expression group underscores the importance of galectin-8 in driving aggressive tumor behaviors. Additionally, GSEA analysis revealed that galectin-8 plays a pivotal role in mediating therapy resistance, particularly in the context of drug-resistant breast cancer subtypes, such as TNBC (Fig. 2D). The enrichment of pathways related to drug resistance in the high galectin-8 expression group suggests that galectin-8 may contribute to resistance mechanisms, potentially by promoting cellular processes like enhanced drug efflux, DNA repair, and evasion of apoptosis. These findings highlight galectin-8 as a potential therapeutic target for overcoming drug resistance in breast cancer. Overall, this analysis emphasizes the critical role of galectin-8 in breast cancer progression, particularly in the aggressive basal-like subtype, and its involvement in driving both EMT and therapy resistance. Targeting galectin-8 could provide new therapeutic strategies to treat resistant forms of breast cancer, improving patient outcomes.

### The expression of galectin-8 in TNBC cell lines

**Figure 3:**
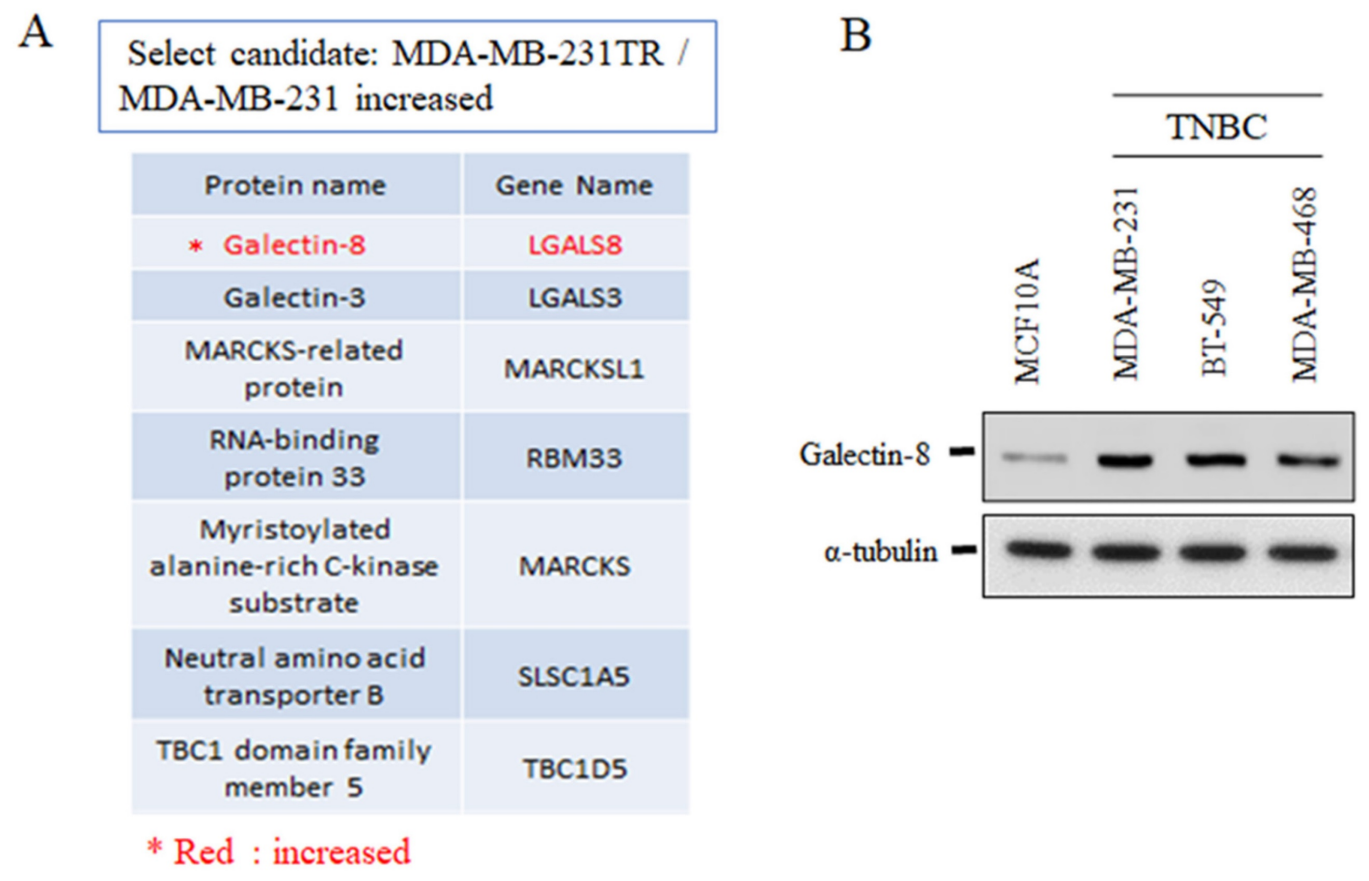
The expression of galectin-8 in TNBC cell lines. (A) Mass spectrometry analysis comparing Tarceva-resistant MDA-MB-231 cells and parental MDA-MB-231 cells. The analysis shows a significant upregulation of galectin-8 in the Tarceva-resistant cell line, suggesting a role in drug resistance mechanisms. (B) Western blot analysis of galectin-8 expression levels in several TNBC cell lines and normal mammary epithelial cells (MCF10A). Galectin-8 expression was markedly elevated in TNBC cell lines compared to MCF10A, indicating its involvement in TNBC progression and aggressiveness.

To further investigate the role of galectin-8 in TNBC, we analyzed its expression in drug-resistant and normal cell lines. Mass spectrometry and Western blotting were employed to evaluate galectin-8 expression in TNBC models, comparing drug-resistant variants and non-tumorigenic mammary epithelial cells. Mass spectrometry analysis revealed that galectin-8 expression was significantly upregulated in the Tarceva-resistant MDA-MB-231 cells compared to the parental MDA-MB-231 cell line (Fig. 3A). The increase in galectin-8 expression in the drug-resistant cell line suggests that galectin-8 may contribute to therapy resistance mechanisms, potentially promoting cell survival and metastasis under treatment pressure. In addition, Western blot analysis was performed to assess galectin-8 expression across several TNBC cell lines and compared to the normal mammary epithelial cell line MCF10A (Fig. 3B). The results demonstrated elevated galectin-8 expression in TNBC cell lines compared to MCF10A, further supporting the hypothesis that galectin-8 plays a critical role in the progression and aggressiveness of TNBC. This increase in expression may also indicate the involvement of galectin-8 in facilitating drug resistance, making it a potential therapeutic target for TNBC treatment. These findings highlight the significant overexpression of galectin-8 in both drug-resistant and TNBC cells, suggesting that targeting galectin-8 could be a promising approach for overcoming resistance in TNBC and improving treatment outcomes for patients with aggressive breast cancer subtypes.

### The expression of galectin-8 in TNBC-tarceva resistant cells

**Figure 4:**
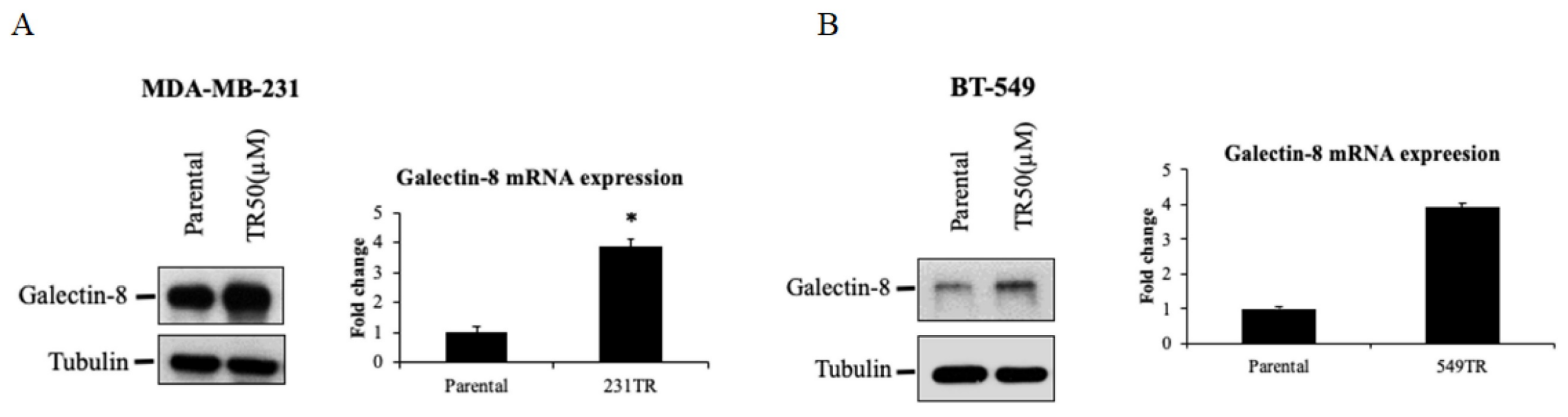
The expression of galectin-8 in TNBC-tarceva resistant cells. (A) Western blot and qPCR analysis of galectin-8 expression in parental MDA-MB-231 cells compared to Tarceva-resistant MDA-MB-231 cells (TR50). Both protein and mRNA levels of galectin-8 are significantly upregulated in the resistant cell line. (B) Western blot and qPCR analysis of galectin-8 expression in parental BT-549 cells compared to Tarceva-resistant BT-549 cells (TR50). Similar to MDA-MB-231, galectin-8 expression is markedly increased in both protein and mRNA levels in the Tarceva-resistant variant. *p<0.05.

To further elucidate the role of galectin-8 in Tarceva-resistant TNBC cells, we examined its expression at both the protein and mRNA levels using Western blot and qPCR analyses. These techniques were applied to evaluate the changes in galectin-8 expression between parental and Tarceva-resistant TNBC cell lines. Western blot analysis was performed to compare galectin-8 protein levels between the parental MDA-MB-231 cells and the Tarceva-resistant variant (TR50) (Fig. 4A). The results showed a marked increase in galectin-8 expression in the Tarceva-resistant cells relative to the parental line, suggesting that galectin-8 may contribute to the resistance mechanism by modulating cellular processes associated with drug evasion. In addition, qPCR analysis was conducted to assess galectin-8 mRNA levels, further confirming the upregulation of this gene in Tarceva-resistant cells. This increase in mRNA levels is consistent with the observed rise in protein expression, indicating that the regulation of galectin-8 occurs at the transcriptional level as well. Similarly, we analyzed another TNBC cell line, BT-549, comparing the parental cells with the Tarceva-resistant TR50 variant (Fig. 4B). Both Western blot and qPCR results revealed a significant upregulation of galectin-8 in the Tarceva-resistant BT-549 cells. These findings further support the hypothesis that galectin-8 plays a critical role in developing drug resistance in TNBC cells, as its expression is consistently elevated across different resistant cell lines. In summary, these results from both Western blot and qPCR demonstrate a consistent upregulation of galectin-8 in TNBC cells that have developed resistance to Tarceva, highlighting its potential as a key regulator of therapy resistance. Targeting galectin-8 may offer a novel therapeutic approach to overcoming drug resistance in TNBC.

### Knockdown of galectin-8 decreases cell proliferation in tarceva-resistant TNBC cells

**Figure 5:**
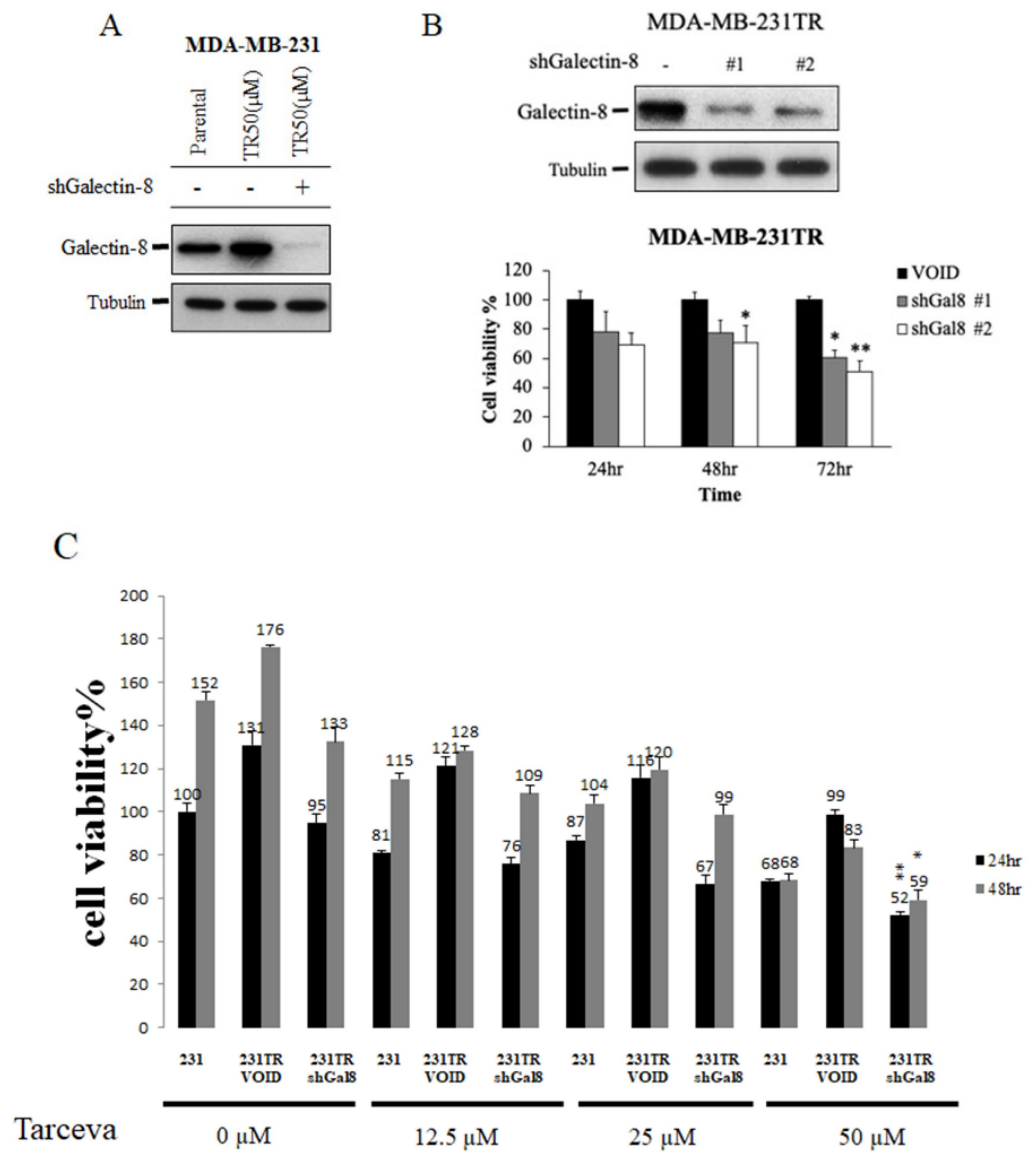
Knockdown of galectin-8 decreases cell proliferation and restores sensitivity to tarceva in TNBC cells. (A) Cell proliferation assay results showing a significant reduction in cell proliferation in Tarceva-resistant MDA-MB-231 (TR50) cells following galectin-8 knockdown using shRNA. (B) Similar cell proliferation reduction was observed in Tarceva-resistant BT-549 (TR50) cells after galectin-8 knockdown, indicating its critical role in promoting cell growth in resistant TNBC cells. “-” indicated negative control. (C) MTT assay comparing MDA-MB-231, Negative control Tarceva-resistant MDA-MB-231TR, and galectin-8 knockdown MDA-MB-231TR cells treated with varying concentrations of Tarceva (5, 10, 25, and 50 µM) for 24 and 48 hours. At 50 µM, galectin-8 knockdown MDA-MB-231TR cells lost their resistance to Tarceva, showing significantly decreased cell viability. *p<0.05, **p<0.01.

To investigate the role of galectin-8 in Tarceva-resistant TNBC cells, we performed knockdown experiments to assess its impact on cell proliferation and drug sensitivity. Using shRNA constructs targeting galectin-8, we successfully reduced its expression in Tarceva-resistant MDA-MB-231 and BT-549 cells. Our goal was to evaluate whether decreasing galectin-8 expression could suppress the proliferation of these resistant cancer cells and restore their sensitivity to Tarceva treatment. After confirming the effective knockdown of galectin-8 via qPCR and Western blot, we conducted cell proliferation assays. The results showed a significant reduction in cell proliferation in Tarceva-resistant MDA-MB-231 cells following galectin-8 knockdown (Fig. 5A). This indicates that galectin-8 is essential for the proliferation of these drug-resistant cells. Similarly, in the Tarceva-resistant BT-549 cells, knockdown of galectin-8 also resulted in a marked decrease in cell proliferation, further supporting the critical role of galectin-8 in maintaining the growth and survival of Tarceva-resistant TNBC cells (Fig. 5B). Additionally, an MTT assay was performed to examine the effect of different concentrations of Tarceva (5, 10, 25, and 50 µM) on MDA-MB-231, Tarceva-resistant MDA-MB-231TR cells, and galectin-8 knockdown MDA-MB-231TR cells. The cells were treated with Tarceva for 24 and 48 hours. At the 50 µM concentration of Tarceva, we observed that knockdown of galectin-8 in the MDA-MB-231TR cells led to the loss of drug resistance, with a significant decrease in cell viability (Fig. 5C). This suggests that inhibiting galectin-8 restores the sensitivity of resistant cells to Tarceva treatment. These findings demonstrate that galectin-8 plays a key role in both the proliferation and drug resistance of TNBC cells. Targeting galectin-8 through knockdown or pharmacological inhibition not only reduces the proliferation of resistant cells but also restores their sensitivity to Tarceva, providing a potential therapeutic approach to overcoming drug resistance in TNBC.

### Knockdown of galectin-8 influences cell migration in tarceva-resistant TNBC cells

**Figure 6:**
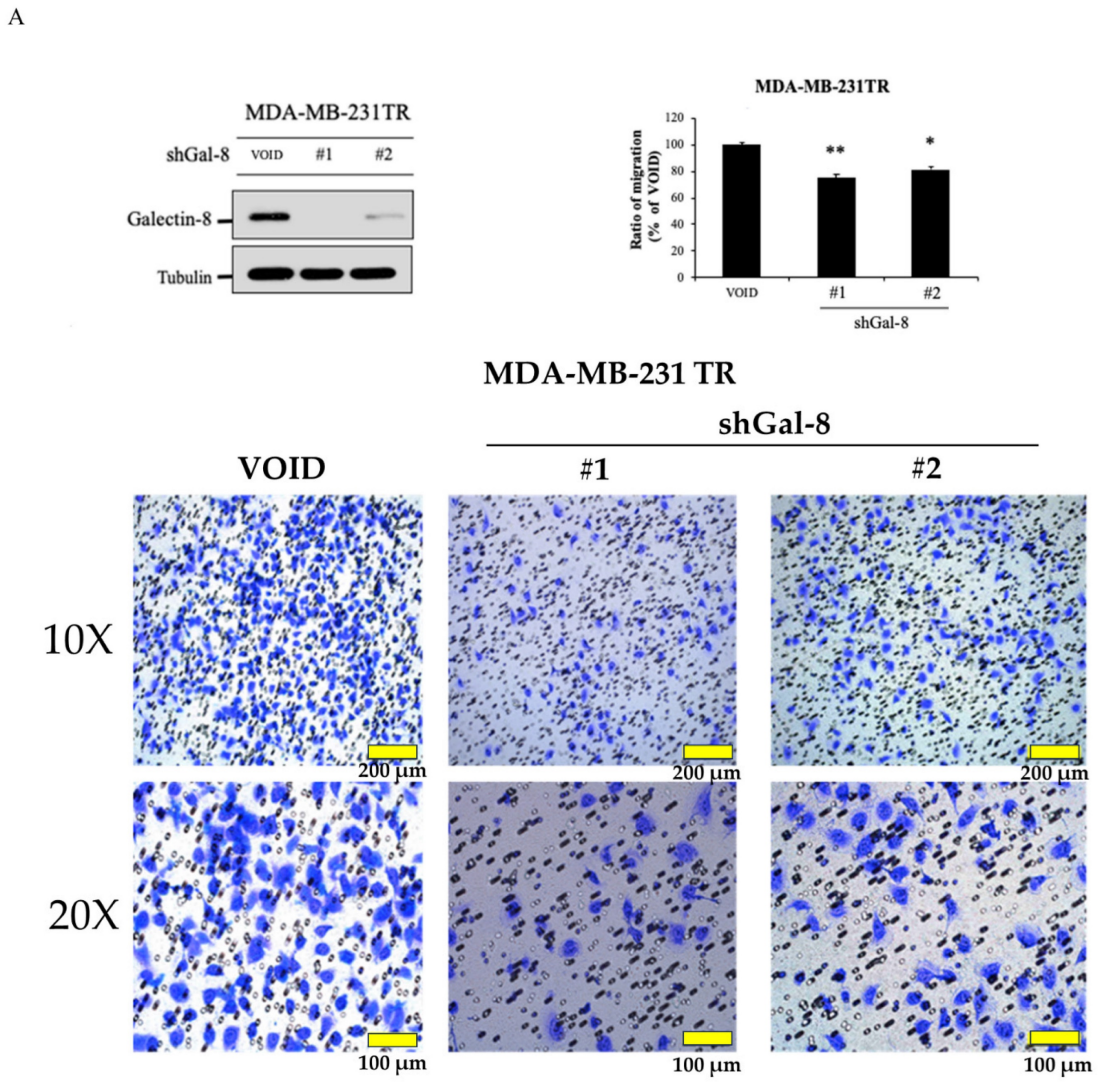

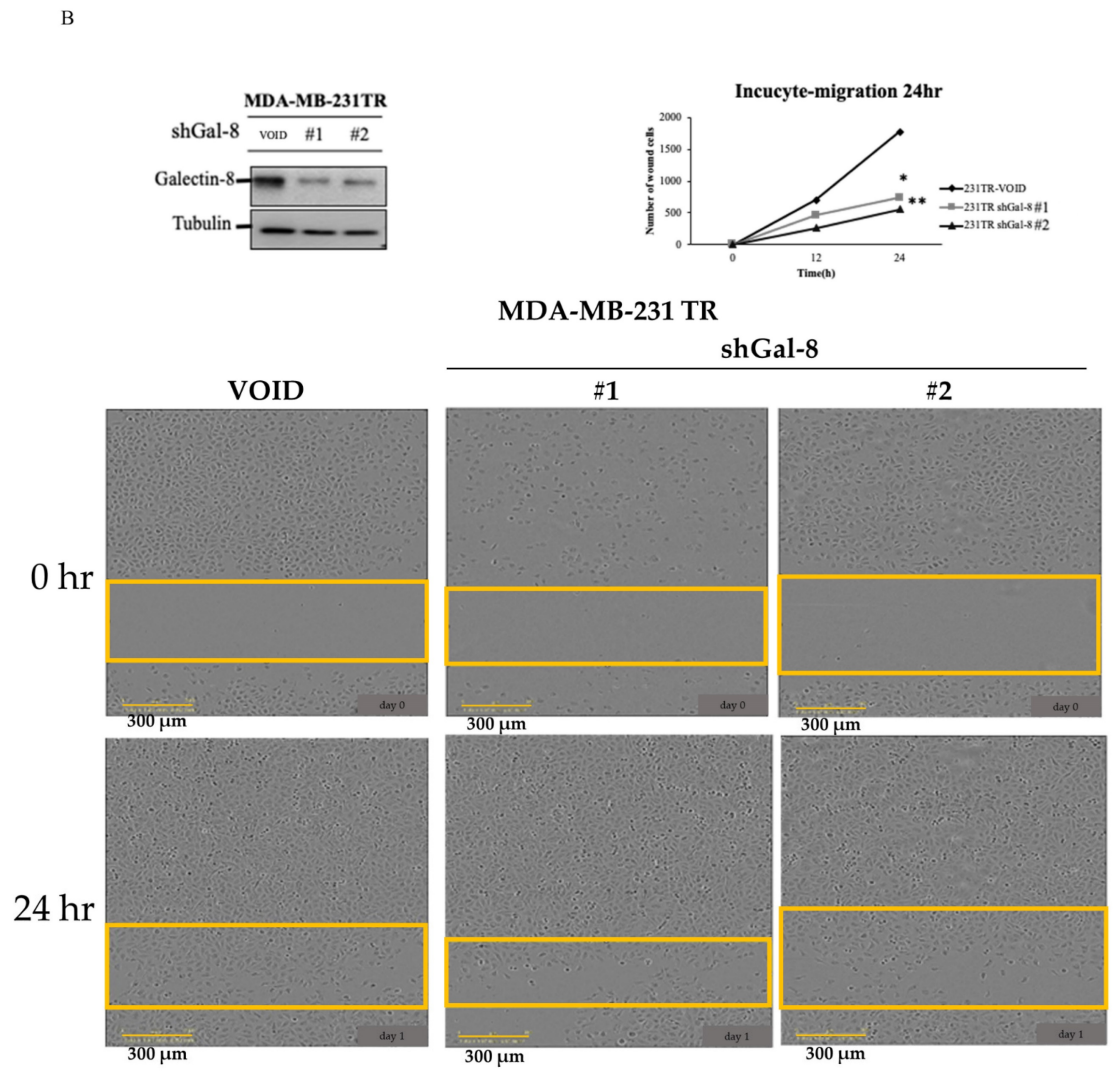
Knockdown of galectin-8 reduces cell migration in tarceva-resistant TNBC Cells. (A) Transwell assay results showing a significant reduction in migration capacity of Tarceva-resistant MDA-MB-231 cells after galectin-8 knockdown compared to the control group. (B) IncuCyte assay demonstrating real-time analysis of cell migration. Galectin-8 knockdown Tarceva-resistant cells showed a marked decrease in migration speed and distance over time compared to control cells. *p<0.05, **p<0.01.

To further investigate the role of galectin-8 in Tarceva-resistant TNBC cells, we assessed its impact on cell migration following galectin-8 knockdown. Two complementary assays, the Transwell assay and the IncuCyte assay, were used to evaluate changes in cell migration behavior. In the Transwell assay, we compared the migration capacity of galectin-8 knockdown Tarceva-resistant MDA-MB-231 cells with that of the control Tarceva-resistant cells. The results revealed a significant reduction in the migration of cells in which galectin-8 was silenced (Fig. 6A). This suggests that galectin-8 plays a critical role in facilitating the migration of Tarceva-resistant TNBC cells, potentially contributing to their invasive potential. The IncuCyte assay further validated the effect of galectin-8 knockdown on cell migration. In this assay, real-time monitoring of cell movement showed that galectin-8 knockdown Tarceva-resistant cells exhibited a substantial decrease in migration speed and distance compared to the control cells (Fig. 6B). This real-time analysis reinforces the finding that galectin-8 is a key driver of cell motility in drug-resistant TNBC cells. Together, these findings indicate that galectin-8 not only supports cell proliferation and therapy resistance in TNBC cells but also promotes their migratory and invasive capabilities. Targeting galectin-8 could provide a dual benefit by reducing both the proliferation and metastatic potential of drug-resistant TNBC cells.

### The EMT marker expression after knockdown of galectin-8 in MDA-MB-231 resistant cells

**Figure 7:**
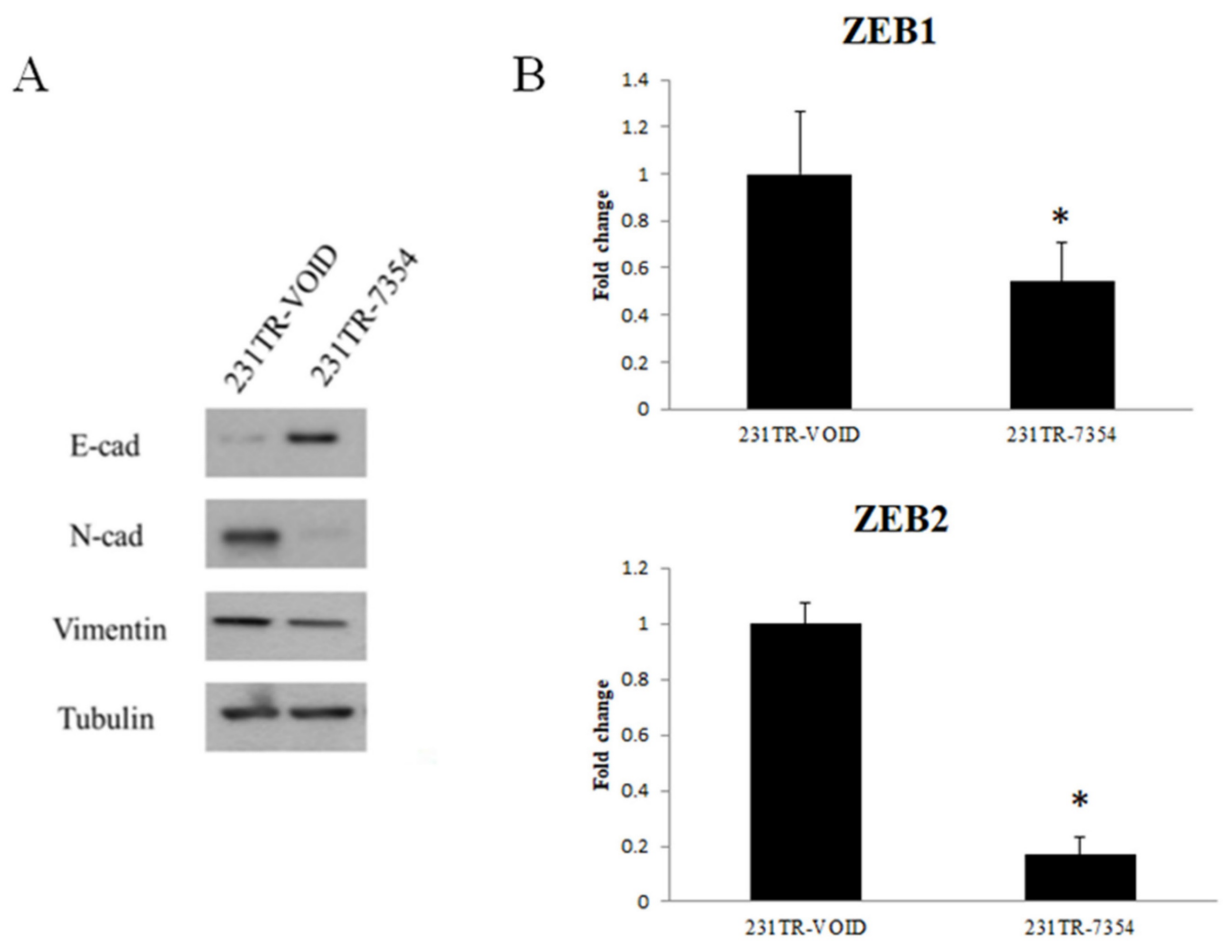
EMT marker expression after knockdown of galectin-8 in MDA-MB-231-resistant cells. (A) Western blot analysis showing increased expression of the epithelial marker E-cadherin and decreased expression of the mesenchymal markers N-cadherin and vimentin following galectin-8 knockdown in Tarceva-resistant MDA-MB-231 cells. These results indicate a reversal of the EMT process. (B) PCR analysis showing reduced mRNA levels of the EMT transcription factors ZEB1 and ZEB2 in galectin-8 knockdown cells, suggesting that galectin-8 is required for the sustained expression of these EMT drivers.

To further investigate the effect of galectin-8 knockdown on EMT, we examined the expression of key EMT markers in Tarceva-resistant MDA-MB-231 cells. Both Western blot and PCR analyses were used to assess the changes in EMT-related proteins and transcription factors after galectin-8 knockdown. The Western blot analysis showed a significant increase in the epithelial marker E-cadherin following galectin-8 knockdown, indicating a shift towards an epithelial phenotype. At the same time, the expression of mesenchymal markers N-cadherin and vimentin was substantially reduced, suggesting a reversal of the EMT process (Fig. 7A). These changes indicate that knockdown of galectin-8 may inhibit the EMT process, which is often associated with decreased invasiveness and metastatic potential in cancer cells. In the PCR analysis, we focused on the expression of ZEB1 and ZEB2, two transcription factors known to regulate the EMT process. Knockdown of galectin-8 resulted in a marked reduction in the mRNA levels of both ZEB1 and ZEB2 (Fig. 7B), further supporting the notion that galectin-8 plays a role in driving the EMT program in Tarceva-resistant cells. These transcription factors are critical for maintaining the mesenchymal state, and their downregulation following galectin-8 knockdown suggests that galectin-8 is necessary for the sustained expression of these EMT drivers. Overall, these results demonstrate that galectin-8 is a key regulator of EMT in Tarceva-resistant MDA-MB-231 cells. By knocking down galectin-8, the expression of epithelial markers is increased while mesenchymal markers and EMT transcription factors (ZEB1, ZEB2) are downregulated, suggesting a potential therapeutic approach for reducing the metastatic behavior of resistant TNBC cells.

## Discussion

TNBC remains one of the most aggressive and difficult-to-treat subtypes of breast cancer, largely due to the absence of hormone receptors and HER2 expression, which limits the use of targeted therapies. Patients with TNBC often rely on chemotherapy, but drug resistance frequently develops, leading to poor outcomes. The recurrence rates and limited therapeutic options make TNBC a significant clinical challenge. While immunotherapy has shown promise for some patients, resistance to systemic therapies continues to be a major hurdle, especially for those with more aggressive, drug-resistant forms of TNBC​ 21-23. In addition to its role in TNBC, galectin-8 has been implicated in other malignancies such as colorectal and ovarian cancers, where it contributes to immune evasion, tumor cell invasion, and metastasis. Studies have shown that galectin-8 promotes immune suppression by recruiting regulatory T cells (Tregs) and myeloid-derived suppressor cells (MDSCs), reducing immune surveillance. These findings suggest that targeting galectin-8 could have therapeutic potential across multiple cancer types, making it a promising candidate for broader oncological treatment strategies 24-26.

Our study provides novel insights into the role of galectin-8 in driving drug resistance, proliferation, and metastasis in TNBC. While previous studies have implicated galectins, particularly Galectin-3, in cancer progression and resistance 27, our findings highlight the specific role of galectin-8 in Tarceva-resistant TNBC cells. Although TNBC lacks HER2 expression, a subset of TNBC cases overexpresses EGFR, and targeting this pathway has shown potential in these subtypes. This justifies the use of Tarceva in our resistance model, as previous studies have demonstrated its efficacy in EGFR-positive TNBC cell lines. For example, Hicks *et al.* demonstrated that EGFR is overexpressed in certain TNBC subtypes, including those that develop brain metastases 28. Additionally, Dent *et al.* highlighted the molecular heterogeneity of TNBC, noting that EGFR expression is present in a subset of patients, which supports the rationale for using EGFR-targeted therapies like Tarceva 29. Elevated levels of galectin-8 were observed in Tarceva-resistant MDA-MB-231 and BT-549 cell lines, both at the mRNA and protein levels, which suggests that galectin-8 is a key player in promoting the aggressive behavior of these cells​.

The knockdown experiments revealed that reducing galectin-8 expression not only decreases cell proliferation but also restores sensitivity to Tarceva, particularly at higher concentrations. This suggests that galectin-8 could serve as a therapeutic target for reversing drug resistance in TNBC. In contrast to other galectins, such as Galectin-3, which have been studied in the context of various cancer subtypes, our findings underscore the unique importance of galectin-8 in regulating drug resistance mechanisms through its influence on cell survival pathways​ 27, 30-32.

Studies have shown that high levels of E-cadherin, an epithelial marker, are correlated with better clinical outcomes in TNBC patients. Loss of E-cadherin expression is often associated with more aggressive and invasive tumor behavior, leading to a poorer prognosis. For example, research demonstrated that the absence of E-cadherin is significantly associated with reduced recurrence-free survival (RFS) and overall survival (OS) in TNBC patients 33, 34. Restoration of E-cadherin expression, as observed following galectin-8 knockdown, may contribute to a less invasive phenotype and improve clinical outcomes in drug-resistant TNBC. Further supporting the role of galectin-8 in tumor progression, knockdown of galectin-8 also significantly reduced cell migration, as evidenced by both Transwell and IncuCyte assays. Additionally, our examination of EMT markers showed that galectin-8 knockdown leads to increased expression of E-cadherin (an epithelial marker) and decreased expression of mesenchymal markers, such as N-cadherin, vimentin, and the EMT transcription factors ZEB1 and ZEB2. This suggests that galectin-8 may play a critical role in maintaining the mesenchymal state of TNBC cells, contributing to their invasiveness and metastatic potential​ 35. To further explore the mechanisms by which galectin-8 modulates EMT in TNBC cells. Oyanadel *et al.* indicated that galectin-8 was shown to drive partial EMT through the activation of FAK/EGFR signaling pathways 10. This mechanistic insight aligns with our observations in the current study, where knockdown of galectin-8 resulted in the reversal of EMT markers. While the development of galectin-8 inhibitors is still in its early stages, alternative therapeutic strategies could focus on combinatory treatments that target both galectin-8 and key pathways associated with EMT. For instance, simultaneous inhibition of galectin-8 and FAK/EGFR signaling could enhance therapeutic efficacy by reducing both drug resistance and metastatic potential. Although this study focuses on *in vitro* models, future investigations will aim to incorporate clinical data from drug-resistant TNBC patients to validate our *in vitro* findings on galectin-8 expression and its role in drug resistance.

Compared to previous studies focusing on other galectins, our work presents a novel perspective by demonstrating that targeting galectin-8 not only disrupts EMT but also reverses drug resistance in TNBC. This adds a new dimension to the growing body of research exploring galectins as therapeutic targets in cancer. Given the limited treatment options for TNBC, particularly for drug-resistant forms, our findings highlight the potential of galectin-8 as a novel therapeutic target.

## Conclusions

In conclusion, targeting galectin-8 represents a promising strategy for overcoming drug resistance and reducing metastasis in TNBC. Further studies are warranted to explore the development of galectin-8 inhibitors and their efficacy in combination with existing therapies, particularly in clinical settings involving drug-resistant breast cancer.
